# Procollagen Triple Helix Assembly: An Unconventional Chaperone-Assisted Folding Paradigm

**DOI:** 10.1371/journal.pone.0001029

**Published:** 2007-10-10

**Authors:** Elena Makareeva, Sergey Leikin

**Affiliations:** Section on Physical Biochemistry, Department of Health and Human Services, National Institute of Child Health and Human Development, National Institutes of Health, Bethesda, Maryland, United States of America; The University of Manchester, United Kingdom

## Abstract

Fibers composed of type I collagen triple helices form the organic scaffold of bone and many other tissues, yet the energetically preferred conformation of type I collagen at body temperature is a random coil. In fibers, the triple helix is stabilized by neighbors, but how does it fold? The observations reported here reveal surprising features that may represent a new paradigm for folding of marginally stable proteins. We find that human procollagen triple helix spontaneously folds into its native conformation at 30–34°C but not at higher temperatures, even in an environment emulating Endoplasmic Reticulum (ER). ER-like molecular crowding by nonspecific proteins does not affect triple helix folding or aggregation of unfolded chains. Common ER chaperones may prevent aggregation and misfolding of procollagen C-propeptide in their traditional role of binding unfolded polypeptide chains. However, such binding only further destabilizes the triple helix. We argue that folding of the triple helix requires stabilization by preferential binding of chaperones to its folded, native conformation. Based on the triple helix folding temperature measured here and published binding constants, we deduce that HSP47 is likely to do just that. It takes over 20 HSP47 molecules to stabilize a single triple helix at body temperature. The required 50–200 µM concentration of free HSP47 is not unusual for heat-shock chaperones in ER, but it is 100 times higher than used in reported *in vitro* experiments, which did not reveal such stabilization.

## Introduction

Type I collagen is the most abundant protein in higher vertebrates. Proper folding of its triple helix is crucial for forming the matrix of bones and other tissues. Folding defects result in severe/lethal bone fragility and deformities (Osteogenesis Imperfecta) [1]–[3]. The triple helix folding follows synthesis of procollagen chains within Endoplasmic Reticulum (ER). Procollagen is a collagen precursor, in which the triple helix is flanked by globular N- and C-terminal propeptides. As with many other proteins, a variety of different chaperone molecules appear to be involved in procollagen folding. Some are general ER chaperones, e.g, calnexin, BiP, GRP94, and PDI [4]. Some are collagen-specific, e.g., HSP47 and prolyl-4-hydroxylase [4], [5]. Some are known mostly for their other functions, but may also act as collagen chaperones, e.g., SPARC [6]–[8]. The most recent additions to the latter family are prolyl-3-hydroxylase (P3H1) and cartilage-associated protein (CRTAP). P3H1 and CRTAP form a tight, ER-resident complex with cyclophilin B known for its peptidyl-prolyl-isomerase activity [9]. Disruptions of this complex by recessive null mutations in CRTAP and P3H1 were recently discovered in several patients with delayed procollagen folding and severe/lethal skeletal deformities reminiscent of Osteogenesis Imperfecta [10]–[12].

The traditional view is that chaperone molecules interact with unfolded and partially folded polypeptide chains, preventing their aggregation and other nonproductive interactions that may result in misfolding [13], [14]. Once the native state is achieved, the protein is believed to be released from its interactions with the chaperone(s). Posttranslational modification of procollagen chains and folding of the globular C-propeptide may follow this pathway [4]. However, folding of procollagen triple helix may not. The best known triple helix chaperone is HSP47, but the molecular mechanism of its action remains controversial [5], [15]–[18]. In particular, HSP47 appears to bind preferentially to the triple helix rather than unfolded chains [17]–[19], opposite to most other ER chaperones.

In the present study, we provide direct experimental evidence suggesting why such non-traditional chaperone action may be required for procollagen triple helix folding. We demonstrate that the main obstacle to achieving the native triple helical conformation is not aggregation or misfolding of procollagen chains but rather intrinsic thermal instability of the native, folded state. Previously we found that mature collagen is thermally unstable at body temperature [20]. We now find that propeptides, divalent ions, and ER-like crowding with proteins do not increase the stability of the triple helix. Moreover, no aggregation of unfolded procollagen chains is induced by crowding with ∼100 mg/ml of proteins that do not specifically interact with collagen. In phosphate buffered saline (PBS) as well as in buffers that mimic some of ER conditions, procollagen triple helix spontaneously folds into its native conformation several degrees *below* but not at body temperature. To fold at body temperature, the triple helix conformation has to be stabilized by over 50 kcal/mol through interactions with chaperone molecules. We hypothesize that these interactions may involve preferential binding of HSP47 and, potentially, other specialized chaperones to the native triple helix. In contrast, traditional binding of chaperones to unfolded chains within the triple helix region will make the folding more rather than less difficult.

## Results

### Thermal denaturation

#### Procollagen vs. collagen

**Figure 1:**
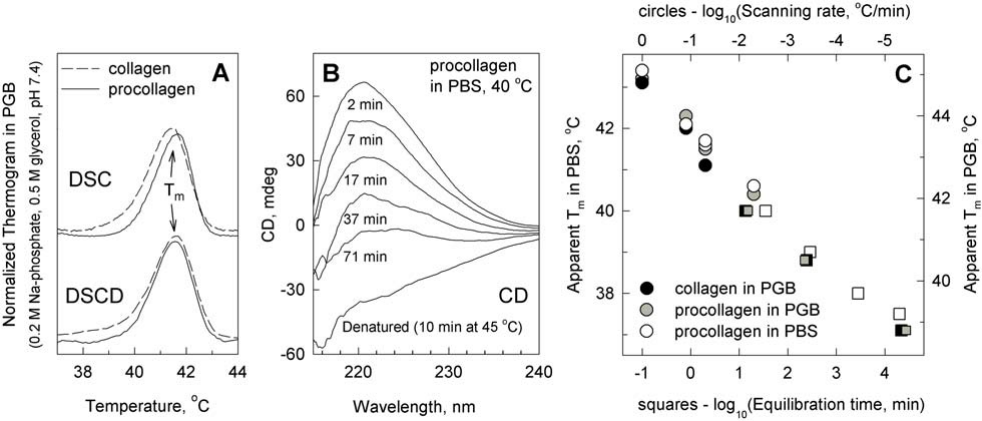
Type I procollagen and collagen have the same thermal stability. A. Denaturation thermograms at 0.05°C/min heating rate in 0.2 M Na-phosphate, 0.5 M glycerol, pH 7.4 (PGB). B. Procollagen denaturation kinetics at 40°C in 0.15 M NaCl, 7 mM Na-phosphate, 1.7 mM K-phosphate, pH 7.4 (PBS). C. Apparent T_m_ of collagen and procollagen in PGB and PBS. In DSC and DSCD measurements (circles), T_m_ was defined from the maximum on the corresponding thermogram (as shown in A). In isothermal CD (squares), the time of 50% denaturation was measured at a given temperature (as shown in B) and the results were plotted as the temperature of 50% denaturation (apparent isothermal T_m_) vs. the corresponding equilibration time.

Comparison of pepsin-treated human type I collagen (hereafter referred to as collagen) with its procollagen precursor revealed no substantial differences in their thermal stability when measured at the same conditions (Fig. 1A,C). The comparison was performed in 0.2 M phosphate, 0.5 M glycerol, pH 7.4 (PGB), to avoid fibrilogenesis of collagen. A single denaturation peak was observed at 1, 0.125, and 0.05°C/min heating rates by DSC as well as at 0.05 and 0.005°C/min by DSCD (Fig. 1A,C). The apparent melting temperature T_m_ at the maximum of the peak was the same for procollagen and collagen, within ±0.3°C reproducibility of the measurements. Furthermore, no differences between procollagen and collagen denaturation half-time at constant temperature were detected by isothermal CD measurements at 38.8, 40.5 and 41.7°C (Fig. 1C). Thus, the N- and C-propeptides do not stabilize collagen triple helix.

Note that the PGB composition simply increases the triple helix stability by 1.7°C compared to physiological conditions [20]. We confirmed this prediction by comparing the apparent T_m_ as well as full DSC and DSCD thermograms of procollagen at different heating rates in PGB and PBS at the same pH (Fig. 1C, grey and white circles). We also confirmed the same buffer effect at different equilibration times in isothermal measurements (Fig. 1C, squares).

#### Unfolding temperature

Similar to collagen [20], [21], procollagen T_m_ depends logarithmically on the heating rate or equilibration time. Indeed, 0.4–0.5°C decrease in the apparent T_m_ was observed upon a two-fold decrease in the heating rate in DSC/DSCD (Fig. 1C, circles). Likewise, 0.4–0.5°C decrease in the temperature at which 50% denaturation occurs was observed upon a two-fold increase in the equilibration time in isothermal experiments (Fig. 1C, squares). Hereafter, we refer to the temperature of 50% denaturation at a given equilibration time as an apparent T_m_ as well. We observed this logarithmic dependence up to the maximum equilibration time of two weeks, at which we were still able to avoid protein degradation. At the two-week equilibration time, procollagen T_m_ in PBS was approximately 37.5°C. Because the logarithmic dependence cannot be extrapolated to infinite equilibration time, we could not determine the equilibrium unfolding temperature of procollagen. In any case, it appears to be lower than normal physiological temperature.

#### Role of divalent ions

**Figure 2:**
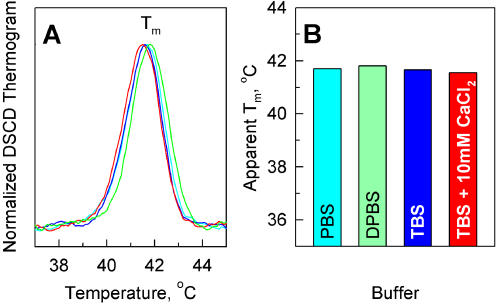
Divalent ions do not affect thermal stability of procollagen. Normalized denaturation thermograms (A) and apparent T_m_ (B) measured by DSCD at 0.05°C/min scanning rate in PBS, DPBS (PBS with 1 mM CaCl_2_ and 0.5 mM MgCl_2_), TBS (50 mM Tris, 150 mM NaCl), and TBS with 10 mM CaCl_2_. All buffers had neutral pH 7.1–7.5. The thermograms in A have the same colors as the corresponding bars in chart B.

In cells, procollagen folds inside ER, which is the main storage compartment for Ca^2+^ [22]. However, we did not observe any detectable effects of either Ca^2+^ or Mg^2+^ on the thermal stability of procollagen (Fig. 2). The thermograms of procollagen melting in PBS, DPBS containing 1 mM CaCl_2_ and 0.5 mM MgCl_2_, TBS (50 mM Tris, 0.15 M NaCl, pH 7.4), and TBS with 10 mM CaCl_2_ were identical.

#### Effect of crowding

ER is also a very crowded environment with ∼100 mg/ml total protein concentration [23]. To mimic such an environment without chaperone proteins, we selected bovine serum albumin (BSA, 66 kD, pI≈4.9) as the primary crowding agent, which is sufficiently thermostable and soluble. Alternatively, as a control for possible BSA-specific artifacts, we used chicken egg white lysozyme (14.6 kD, pI≈11) or human immunoglobulin G (IgG, 150 kD, pI≈5.8–7.3), which have different molecular weights, charges and sequences and are just as stable and soluble as BSA.

**Figure 3:**
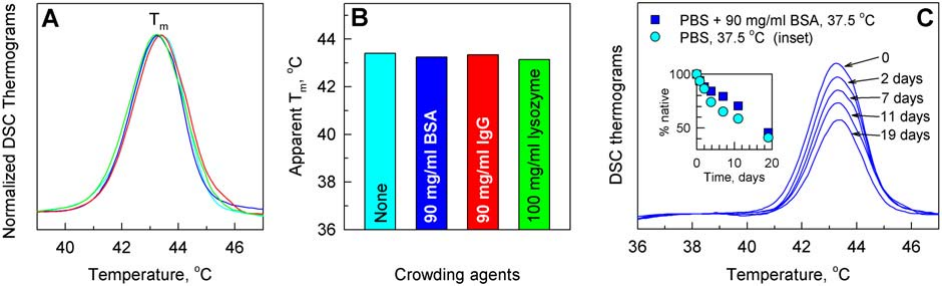
ER-like molecular crowding with nonspecific proteins does not affect procollagen thermal stability. A and B. Normalized DSC thermograms (A) and apparent T_m_ (B) at 1°C/min heating in PBS without and with 90 mg/ml BSA, 90 mg/ml IgG, or 100 mg/ml lysozyme (the thermograms in A and the corresponding bars in B have the same colors). C. Unfolding kinetics of procollagen at 37.5°C. In PBS with 90 mg/ml BSA, native procollagen fractions (inset, squares) were measured from the area under 1°C/min DSC thermograms of sample aliquots (blue tracings). In PBS without BSA, native procollagen fractions were measured from CD as shown in Fig. 1B.

In DSC experiments at 1 and 0.05°C/min heating rate, we did not observe any effects of 90 mg/ml BSA, 100 mg/ml lysozyme, or 90 mg/ml IgG on the stability of procollagen triple helix (Fig. 3A,B). Also, DSC thermograms measured with 0, 10, 37, 65, and 90 mg/ml BSA at 1°C/min heating rate were all identical (data not shown). Because of their high thermal stability (T_m_>65–70°C), BSA, lysozyme or IgG did not contribute to DSC thermograms below 50°C so that procollagen denaturation could be easily detected despite their large concentrations. We also did not observe any significant effects of 90 mg/ml BSA in isothermal procollagen denaturation experiments at 37.5°C (Fig 3C, inset). To monitor the extent of denaturation, we collected aliquots after different time intervals and measured their DSC thermograms at 1°C/min heating rate (Fig. 3C). We used the area under each DSC peak as a measure of the amount of native triple helices in the sample. To reduce degradation of the denatured procollagen by residual proteases potentially present in the sample, we added 0.1 mM EDTA and 0.05 mM PMSF to the buffer. However, the actual extent of the degradation in this experiment could not be controlled by gel electrophoresis due to the high BSA concentration.

### Refolding

#### Procollagen vs. collagen

Collagen refolding is commonly monitored by recovery of the characteristic triple helix CD signal. However, the same CD signal can be recovered in a non-native conformation, e.g., with improper chain register and gelatin-like participation of the same chain in several different triple helices. Indeed, despite the recovery of up to 60% of the CD signal, refolding of pepsin-treated type I collagen produced mostly gelatin-like triple helices and only a small fraction of full-length, pepsin-resistant helices [20]. Furthermore, the full-length helices were mostly composed of α1(I)_3_ homotrimers and α2(I)_3_ homotrimers with only a tiny fraction of normal α1(I)_2_α2(I) heterotrimers.

Therefore, to monitor the recovery of the native procollagen conformation, after each refolding experiment we measured a denaturation thermogram of the sample by DSCD or DSC. The denaturation thermograms can distinguish not only gelatin-like conformations [20], chain register disruptions [24] and chain composition [20] but also small folding defects, such as those introduced by substitutions of obligatory glycines [25] and even some non-glycine substitutions [26].

**Figure 4:**
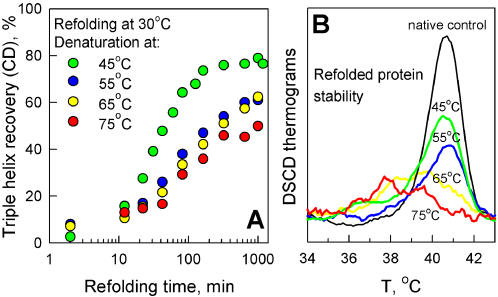
Native structure of procollagen triple helix spontaneously refolds at 30°C after mild denaturation. A. Kinetics of triple helix recovery at 30°C monitored by CD at 223.8 nm in 0.1 mg/ml procollagen solution in PBS after 10 min denaturation at indicated temperatures. B. DSCD thermograms (0.05°C/min) of a native control sample and the refolded procollagen solutions after 10 hour equilibration. The native control has a single, narrow peak at ∼41°C. Additional peaks at lower temperature in the refolded samples originate from shorter, less stable, gelatin-like helices.

Figure 4A shows the refolding kinetics of human type I procollagen in PBS, pH 7.4 at 30°C measured by CD after 10 min denaturation of the triple helix at 45, 55, 65, and 75°C. The refolding was faster after the equilibration at 45°C and similar after the equilibration at 55, 65, and 75°C. However, DSCD thermograms of these samples (Fig. 4B) revealed that little or no procollagen refolded into the native conformation after the equilibration at 65 or 75°C. The DSCD peak at ∼41°C represents denaturation of native procollagen, as indicated by the normal control. The peaks at lower temperatures represent denaturation of less stable, shorter, gelatin-like helices formed as a result of improper refolding [20]. Apparently, higher temperature denaturation resulted in irreversible conformational changes within the C-propeptide, which remained in the native conformation after 10 min at 45°C (see Discussion). Note that these changes were not accompanied by disruption of inter-chain disulfide bonds, as indicated by gel electrophoresis under non-reducing conditions. Based on these observations, all further refolding experiments in CD were performed after the triple helix denaturation at 45°C for 10 min.

From the areas under the 41°C DSCD peak of the initial and renatured procollagens, we estimated that 60–70% of molecules refolded into the native triple helical conformation at 30°C after 10 min at 45°C. At the same time, gel electrophoresis revealed that the initial sample contained 30–35% molecules in which C-propeptides were cleaved from one, two or all three procollagen chains. Our observations were, therefore, consistent with complete refolding of the native triple helical structure in all intact molecules.

#### Refolding temperature

**Figure 5:**
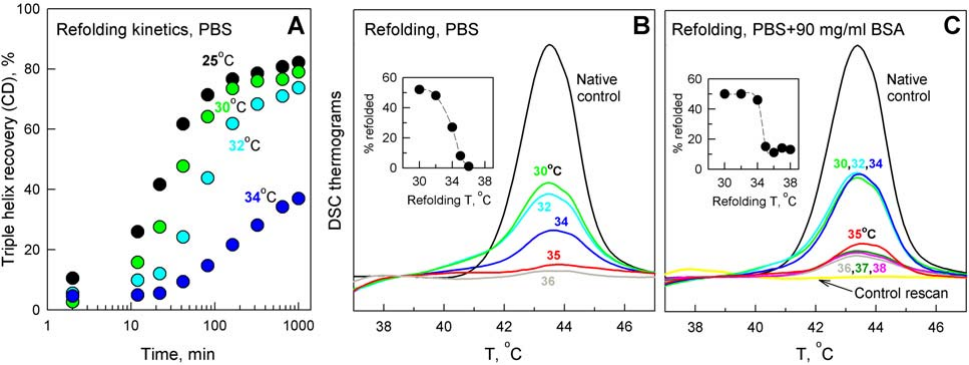
Procollagen triple helix spontaneously refolds below but not above 34°C. A. Kinetics of 0.1 mg/ml procollagen refolding in PBS after 10 min denaturation of the triple helices at 45°C (monitored by CD as in Fig. 4). B and C. Native triple helix refolding in PBS without (B) and with 90 mg/ml BSA (C) after an initial DSC scan from 25 to 50°C at 1°C/min. The fraction of refolded native procollagen (insets) was measured from the area under the DSC thermograms (colored tracings) after overnight equilibration in the DSC instrument at indicated temperatures following the initial denaturation scan (native control). A second scan without the overnight equilibration is shown by the yellow line in C.

We observed complete refolding of native triple helices in all intact molecules at 25, 30 and 32°C in PBS with the renaturation half-time of ∼20, 35 and 70 min, correspondingly (Fig. 5A). At 34°C, the refolding half-time was ∼140 min and only ∼25% of intact molecules refolded into the native structure based on DSCD measurements (data not shown). We observed no significant refolding at 35 and 36°C. All samples were tested by gel electrophoresis to confirm that no degradation or propeptide cleavage occurred during the refolding. Whenever some degradation was observed due to contamination by residual proteases, the experiment was repeated.

Thus, the triple helix formation in PBS is favorable below and unfavorable above 34°C. Above 34°C, procollagen chains appear to remain unfolded in solution rather than undergo irreversible aggregation. In particular, we did not detect any spectroscopic indications of secondary structure formation or aggregation (turbidity or circular dichroism changes) upon equilibration at 37°C. Furthermore, we observed refolding of native procollagen triple helices when 10 min denaturation at 45°C was followed by two-hour equilibration at 37°C with subsequent refolding at 32°C.

Essentially the same results were obtained by DSC (Fig. 5B) using a protocol designed for comparison with renaturation in a crowded environment. In DSC experiments, 0.1 mg/ml procollagen solution was loaded into a calorimeter and denatured by scanning from 25 to 50°C at 1°C/min heating rate. The calorimeter was programmed to begin cooling the sample cell to the desired refolding temperature immediately after the end of the heating cycle. The sample was equilibrated overnight in the calorimeter at the designated refolding temperature, rescanned from 25 to 50°C next morning to determine the extent of refolding, and discarded. Each refolding experiment was performed with a freshly prepared procollagen solution. Approximately 50% of all molecules (70–80% of intact molecules) refolded into the native conformation at 30 and 32°C (Fig. 5B, inset). The refolding fraction was approximately three times smaller at 34°C and virtually no refolding was observed at or above 35°C, consistent with the DSCD observations. Slightly lower refolding fractions in DSC experiments were most likely related to C-propeptide unfolding in some of the molecules due to heating to 50 rather than 45°C (c.f., Fig. 4B).

#### Role of divalent ions

The kinetics and extent of procollagen refolding were similar in PBS, DPBS, TBS, and TBS with 10 mM CaCl_2_ at the same ionic strength and pH (7.1–7.4), within the 5-10% reproducibility of the measurements (data not shown). Thus, Ca^2+^ and Mg^2+^ ions do not appear to affect both unfolding and refolding of procollagen.

#### Effect of crowding

In DSC refolding experiments, 90 mg/ml BSA did not have a significant effect on procollagen refolding (Fig. 5C). As in PBS without BSA, ∼50% of all molecules refolded into the native conformation at 30–34°C. The refolding fraction sharply dropped at higher temperatures to ∼10% at 35–38°C (Fig. 5C, inset). Because BSA had only weak effects and each measurement required a large amount of procollagen, the refolding experiments were not repeated with lysozyme or IgG. The residual 10% refolding was most likely a kinetic artifact of incomplete unfolding in highly viscous BSA solutions (see Discussion). In any case, however, our observations suggest that crowding by globular proteins, which do not exhibit specific interactions with collagen, only weakly (if at all) affects procollagen folding. Such a crowding is not sufficient to ensure the effective folding in ER at physiological temperature.

## Discussion

### *In vitro*, procollagen triple helix is unstable at body temperature

The present study demonstrates that the triple helix is equally unstable at body temperature in mature type I collagen and in its procollagen precursor (Fig. 1C). The C- and N-propeptides, physiological salt, divalent ions or crowding with up to 100 mg/ml of globular proteins do not increase the triple helix stability. At 37.5°C, the triple helix of human procollagen unfolds with the half-time ∼2 weeks. The unfolding half-time decreases two fold for every 0.4–0.5°C increase in the temperature.

The slow denaturation at 37.5°C results from thermal instability of the native triple helix (*N*) with respect to reversible unfolding (*N*↔*U*) rather than slow, irreversible accumulation of unfolded/misfolded chains in aggregates (*N*↔*U*→*A*). Indeed, procollagen molecules with intact C-propeptides appear to be fully capable of spontaneous refolding into the native conformation below 34°C. Above 35°C, however, no triple helix refolding is observed and procollagen chains appear to remain unfolded in solution without aggregating (Fig. 5).

Note that the ability of type I procollagen to refold into the native conformation was first demonstrated by slow dialysis from a urea solution [27]. Without urea, a significant fraction of molecules was found to refold into non-native triple helices at 20°C [28]. However, later studies revealed suppression of such misfolding above 30°C due to lower stability of the resulting helices [20]. In the present study, we find that almost all intact procollagen refolds into the native conformation at 30–32°C after 10 min denaturation of the triple helix at 45°C. The refolding fraction decreases at higher denaturation temperature (Fig. 4) and appears to be limited primarily by irreversible changes in the C-propeptide. At the same conditions, C-propeptides purified from human type I collagen exhibit a broad, irreversible transition between 40 and 60°C (Kuznetsova and Leikin, unpublished results), suggesting that they may indeed remain in the native conformation after 10 min equilibration at 45°C but not at higher temperatures.

### Crowding with globular proteins does not stabilize the triple helix and does not affect aggregation of unfolded procollagen chains

10–90 mg/ml BSA, 100 mg/ml lysozyme or 90 mg/ml IgG have no effect on the apparent T_m_ of procollagen triple helix at either fast or slow denaturation (Fig. 3). Apparently, excluded volume effects and other interactions of procollagen with these proteins are negligible compared with the energies involved in triple helix unfolding. For instance, electrostatic interactions with charged BSA (pI≈4.9) or lysozyme (pI≈11) may be weak because of the low net charge of the triple helix (pI∼6–7) at neutral pH and sufficiently short screening length in physiological salt.

The maximum temperature of procollagen refolding is also minimally (if at all) affected by 90 mg/ml BSA. In DSC experiments, the fraction of refolded triple helices decreased from ∼50% at 30–32°C to almost zero above 35°C without BSA and from ∼50% at 30–34°C to ∼10% at 36–38°C with 90 mg/ml BSA. The same refolding fraction below 34°C with and without BSA suggests not only that BSA does not affect the triple helix folding but also that it does not promote aggregation of unfolded chains.

The residual 10% refolding above 35°C is not caused by generalized stabilization of the triple helix by BSA. Otherwise, it would strongly depend on the temperature above 35°C, as it does between 34 and 35°C. One possible explanation of this apparent refolding is the following kinetic artifact. High viscosity of 90 mg/ml BSA solution may trap procollagen molecules in some intermediate, partially unfolded state after the initial fast heating cycle. A fraction of these molecules may return into the native conformation after overnight equilibration at 35–38°C, even if an unfolded conformation is more energetically favorable at these temperatures. Another explanation may be that ∼10% of procollagen molecules are more susceptible to interactions with and significant stabilization by BSA, e.g., due to Hyl glycosylation at different, less common sites.

### Cells are capable of stabilizing procollagen triple helix by over 50 kcal/mol

Cells are not only capable of folding procollagen up to 40°C [29], but they are also capable of folding mutant triple helices that have reduced thermal stability [30]. Since no spontaneous procollagen refolding occurs above 34°C *in vitro*, the triple helix stability within ER must be at least 5°C higher than in physiological saline. To raise the maximum refolding temperature by *δT* from *T*_0_≈307 K (34°C) in PBS, procollagen triple helix must be stabilized at *T = T*_0_ by *δ*(Δ*G*) = *δT*Δ*H*/*T*_0_ (see Eq. (6) in Methods), where Δ*H*≈3400 kcal/mol is the unfolding enthalpy of human procollagen in PBS measured from the area under the DSC thermograms shown in Fig. 1A. Because of the very large unfolding enthalpy, Δ*H*/*T*_0_≈11 kcal mol^−1^ deg^−1^, procollagen folding at 38–40°C requires triple helix stabilization by 45–65 kcal/mol.

This stabilization is not associated with divalent ions or nonspecific crowding effects of other proteins. In principle, we cannot exclude a contribution of various small solutes (amino acids, sugars, etc.), which may be present in ER in millimolar concentrations. However, unless these solutes are many orders of magnitude more efficient than glycerol, which increases the apparent T_m_ by 0.0008°C/mM [20], it is unlikely that they have a significant effect on the maximum folding temperature. We also cannot exclude potential triple helix stabilization due to procollagen anchoring at the ER membrane [31]. The corresponding confinement effect, however, is unlikely to increase *δ*(Δ*G*) by much more than *RT* ln(2)≈0.4 kcal/mol (due to ∼2-fold reduction in the volume accessible to unfolded procollagen chains). Most likely, procollagen folding in ER involves more specific action of some chaperone molecules, which increase the triple helix stability by preferential binding to it.

Note that procollagen T_m_ was reported to be ∼3°C higher in cell lysates and cell culture media compared to 0.4 M NaCl, 0.1 M Tris [32]. This difference, however, may not be related to the stabilization discussed above. Indeed, NaCl reduces the triple helix T_m_ by 3.8±0.1°C/M [20]. Hence, the 0.4 M NaCl concentration alone may be responsible for about half of the observed difference. In addition, both cell lystaes and cell culture media may contain procollagen aggregates, in which the triple helix may have higher T_m_. Such aggregates form in Golgi (see, e.g., [33]) and they may not completely dissociate upon secretion into cell culture media [34].

### HSP47 may be responsible for triple helix stabilization in ER

One collagen-specific chaperone is HSP47, but the mechanism of its action remains controversial. No increase in the apparent collagen T_m_ is induced by ∼1 µM HSP47 in 0.4 M NaCl, 50 mM Tris [15]. Some authors argue that HSP47 binds equally well to folded and unfolded collagen chains [35], [36]. Hence, many believe that HSP47 is responsible for preventing aggregation and secretion of partially folded and misfolded molecules rather than for triple helix stabilization [5], [15], [37].

More recent data, however, provide a compelling evidence of preferential HSP47 binding to triple helices compared to weak or negligible binding to unfolded chains [19]. Note that this is not inconsistent with the well-documented ability of HSP47 to bind to gelatin [35], [38] since a significant fraction of gelatin chains may be folded into triple helices [39]. Thus, we decided to evaluate the extent of the triple helix stabilization expected for type I procollagen based on the binding constants reported in [40].

**Figure 6:**
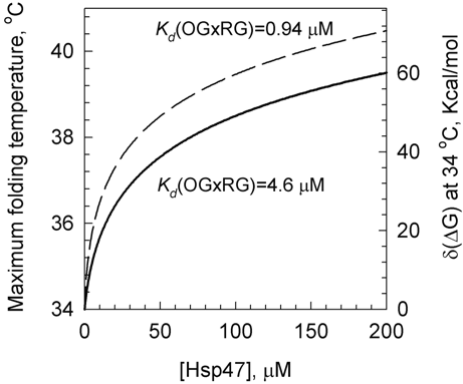
HSP47 may allow procollagen triple helix folding at normal and elevated body temperatures. The free energy of triple helix stabilization *δ*(Δ*G*) and the corresponding maximum triple helix folding temperature *T*_0_+*δT* were calculated from Eqs. (5),(6) based on the HSP47 binding sites and dissociation constants reported in [40], as described in Methods (Theoretical analysis). Two possible values of the dissociation constant *K_d_*(OGxRG) = 4.6 µM (solid line) and *K_d_*(OGxRG) = 0.94 µM (dashed line) were used to account for the uncertainty in HSP47 binding at OGxRG, where x is a variable amino acid.

Straightforward thermodynamic analysis (see Methods) predicts a relationship of *δ*(Δ*G*) and the maximum triple helix folding temperature *T*_0_+*δT* with the HSP47 dissociation constants at different sites on folded and unfolded procollagen chains, Eqs. (5),(6). After substitution of the dissociation constants reported in [40] and *T*_0_≈307 K measured above into Eqs. (5),(6), we calculated *T*_0_+*δT* as a function of HSP47 concentration without any adjustable parameters. The results shown in Fig. 6 indicate that ∼50 µM (2.5 mg/ml) HSP47 will allow procollagen folding up to 38°C and 100–200 µM (5–10 mg/ml) HSP47 will allow procollagen folding up to 40°C. Such concentrations of a heat-shock chaperone would not be unusual for ER, e.g., the concentration of GRP94 (a member of the Hsp90 family) was estimated as 10 mg/ml [41]. On a cautionary note, however, we used the dissociation constants measured in 0.4 M NaCl, 50 mM Tris for relatively short peptides with a limited set of HSP47 recognition sequences. The actual constants for type I collagen triple helix in ER may be different, affecting the HSP47 concentrations required for the triple helix stabilization.

Not only this estimate appears to be consistent with the range of temperatures at which cells are capable of folding procollagen, but it also suggests why no thermal stabilization of collagen by HSP47 was observed *in vitro* in [15]. At the corresponding total concentrations of collagen (∼0.1 µM) and HSP47 (∼1 µM), the expected *δ*(Δ*G*) is less than 5 kcal/mol and the expected *δT* is less than 0.5°C, which is difficult to detect.

We believe that our results support the hypothesis [18], [42] that HSP47 assists procollagen folding by stabilizing the triple helix. Note that HSP47 may not be the only molecule that has this function. For instance, SPARC family proteins may have a similar chaperone activity [6]–[8]. Slower triple helix folding in null mutations of P3H1 or CRTAP [10]–[12] may also indicate their involvement, more likely as a part of the complex with cyclophilin B [9], which is known to be involved in triple helix folding as a peptidil-prolyl cis-trans isomerase [43], [44].

### Implications for general mechanisms of protein folding

In contrast to HSP47, most other chaperones destabilize native proteins by preferential binding to unfolded, partially unfolded, and misfolded polypeptide chains. They guard against various folding traps such as non-productive aggregation and guide/catalyze proper folding steps [13], [14]. Once the native conformation is achieved, the chaperone's job is finished and it dissociates, allowing the protein to follow its own destiny.

However, such destabilization of the native conformation makes folding of marginally stable or unstable proteins such as procollagen more difficult or impossible. In this case, the native conformation may have to be stabilized by another type of chaperone molecules. In particular, to achieve the stabilization of procollagen at body temperature, over twenty HSP47 molecules may have to bind to a single triple helix. They do not dissociate from the triple helix once its folding is complete. Instead, they guide the folded procollagen into Golgi, where they dissociate due to lower pH before being transported back to ER [5], [16], [45].

Are these just peculiar features of procollagen folding or is this a more general chaperone-assisted folding paradigm? While we do not know the exact answer to this question, we believe that the implications of our other findings do extend beyond procollagen.

Based on hard-sphere models, some authors argue that molecular crowding inside cells may promote non-productive aggregation of unfolded protein chains and, at the same time, enhance folding by favoring more compact states of the chains [46], [47]. Experimental data, however, do not provide unequivocal evidence for these or other molecular crowding effects in protein folding [48]–[52]. One source of the problem may be in the common choice of polyethylene glycol, dextran and Ficol as the crowding agents. Not only the concentration dependence of the activity of these polymers is very different from that of hard spheres or proteins, but their interactions with proteins may also be more complex. The present study provides an example of ER-like crowding by proteins which do not exhibit specific interactions with procollagen. At least in this case, we find no indications of either stronger chain aggregation or substantially enhanced folding. At the same time, our attempts to utilize polyethylene glycol, dextran or Ficol produced such strong procollagen aggregation that refolding experiments could not be performed at all. These results do not mean that molecular crowding in cells will not affect aggregation and folding of other proteins or that molecular crowding will not affect interactions of procollagen with its chaperone proteins. But, they do indicate that better understanding of intracellular crowding effects may require some caution in the choice of appropriate crowding agents.

## Materials and Methods

### Experimental procedures

#### Cell Culture

Normal skin fibroblast cultures (CRL-2127, ATCC) were used for large-scale preparations of normal procollagen. Fibroblasts were cultured in Dulbecco's Modified Eagle Medium (DMEM, Invitrogen) containing 10% fetal bovine serum (Invitrogen) and 2 mM glutamine in the presence of 5% CO_2_. When cells became confluent, fresh DMEM supplemented with 2 mM glutamine, 0.1% fetal bovine serum and 50 µg/ml ascorbate was added to the cell cultures. The medium was harvested at 24 h intervals for 3 days and the fresh medium containing ascorbate was replenished daily. After harvesting, the medium was buffered with 100 mM Tris-HCl pH 7.4 and cooled to 4°C. Protease inhibitors were added to the following final concentrations: 25 mM EDTA, 0.2% NaN_3_, 1 mM phenylmethanesulfonyl fluoride (PMSF), 5 mM benzamidine, and 10 mM N-ethylmaleimide (all from Sigma). The medium was filtered using glass microfibre filters GF/A (Whatman) Procollagen was precipitated with 176 mg/ml ammonium sulfate at 4 °C overnight followed by centrifugation at 21000 g for 1 h in L8-70M Ultracentrifuge (Beckman).

#### Procollagen purification

Type I procollagen was purified by ion exchange chromatography in an Akta-Purifier (GE Healthcare) on two 1.6×5 cm columns of DEAE cellulose (DE52, Whatman), as described [53]–[55]. On the first column, the mixture was loaded in 2 M urea, 0.15 M NaCl, 0.1 M Tris-HCl (pH 7.4) and eluted by the same buffer. On the second column, the mixture was loaded in 2M urea, 0.1 M Tris-HCl (pH 8.6) and eluted by NaCl gradient in this buffer. Fractions were analyzed by UV absorbance at 215, 254, and 280 nm and by SDS/PAGE on 4–12% Bis-Tris gels mini-gels with MOPS running buffer (Invitrogen). Fractions containing procollagen were pooled together. After dialysis against PBS, procollagen was precipitated with 176 mg/ml ammonium sulfate and redissolved in PBS at desired concentration. To avoid procollagen degradation, all buffers contained protease inhibitors (20 mM EDTA, 1 mM PMSF, 5 mM benzamidine, and 10 mM N-ethylmaleimide).

#### Differential Scanning Calorimetry (DSC)

DSC scans were performed at 0.05, 0.125 and 1°C/min heating rates in a Nano II or Nano III DSC instrument (Calorimetry Sciences Corporation, Lindon, UT). The apparent melting temperature was defined at the maximum of the melting peak after the baseline subtraction. To mimic the crowded environment of Endoplasmic Reticulum, DSC scans were also performed in the presence of BSA (A7638, Sigma), IgG from human serum (I4506, Sigma) and lysozyme from chicken egg whites (L6876, Sigma).

#### Circular Dichroism (CD)

CD spectra from 215 to 240 nm were measured in a J810 (Jasco, Easton, MD) spectropolarimeter equipped with a 150W xenon lamp and a PFD-425S thermoelectric temperature controller (Jasco, Easton, MD). To avoid protein damage, the shutter was always closed between the measurements; so that cumulative UV exposure never exceeded 10 min. The kinetics of triple helix formation and denaturation was evaluated from the change in ellipticity at 223.8 nm, as described [56].

#### Differential Scanning Circular Dichroism (DSCD)

The solution ellipticity at 223.8 nm was measured from 33 to 45°C with 0.1°C step and 2 or 20 min equilibration time at each temperature. To minimize UV damage, the shutter was open only during the 7 s ellipticity measurements at each temperature (<15 min total UV exposure). Each DSCD thermogram was calculated as the temperature derivative of the ellipticity.

### Theoretical analysis

To evaluate the expected effect of HSP47 on folding and thermal stability of procollagen, we calculated the change *δ*(Δ*G*) in the unfolding free energy Δ*G* caused by preferential ligand binding,
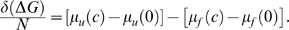
(1)Here *N* is the number of procollagen molecules, *c* is the ligand concentration, and *μ_u_* and *μ_f_* are the chemical potentials of unfolded and folded (native) procollagen, respectively. We assumed that HSP47 binding is not cooperative and that native procollagen has *n* independent binding sites *i* = 1,2,…*n* while unfolded procollagen has *m* binding sites *j* = 1,2,…*m*. Each binding site *i* has the dissociation constant *K^i^_f_* and each binding site *j* has the dissociation constant *K^i^_u_*. The occupation probabilities *θ^i^_f_* and *θ^j^_u_* for each of the sites are given by
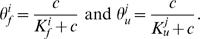
(2)In equilibrium, the chemical potentials of molecules with different number of occupied binding sites are the same and equal to the chemical potential of procollagen with no bound HSP47. The concentration of the latter procollagen molecules is given by
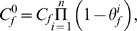
(3)where *C_f_* is the total concentration of all native procollagen molecules. Then, the dependence of the chemical potential of native procollagen on the concentration of free HSP47 can be calculated from
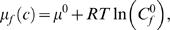
(4)where μ^0^ is the standard chemical potential of procollagen without HSP47. Substitution of Eqs. (2),(3) into Eq. (4) and similar calculation of *μ_u_*(*c*) yield
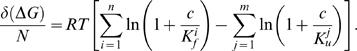
(5)Note that Eq. (5) can be easily adapted for cooperative ligand binding by the corresponding modification of Eqs. (2). In our opinion, however, the reported data for HSP47 binding to triple helical peptides [17], [19], [40] and to collagen [15], [35] do not support the cooperative binding model proposed in [15].

We defined the maximum folding temperature of procollagen as the temperature at which 50% of the triple helices would fold given an infinite equilibration time, i.e., Δ*G* = 0 (assuming completely reversibility of folding/unfolding processes). The effect of ligand binding on this temperature can be evaluated from the following relationship
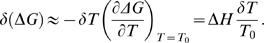
(6)Here *T*_0_ is the maximum folding temperature without and *T*_0_+*δT* is the maximum folding temperature with the ligand; and Δ*H* is the unfolding enthalpy at *T*_0_ without the ligand.

We calculated the expected *δ*(Δ*G*) and *T*_0_+*δT* in the presence of HSP47 from Eqs. (5),(6) based on the HSP47 dissociation constants *K^i^_f_* and *K^j^_u_* for yGxRG sequences reported in [40], where y and x indicate variable residues. Since *K^j^_u_*≥1 mM [40], we neglected the corresponding terms in Eq. (5). We then calculated *δ*(Δ*G*) and *δT* based on the α1(I) sequence. Although *K^i^_f_* were measured in [40] for peptides with three identical chains, most α1(I) and α2(I) recognition sites are aligned with each other and sequence variation in one of the chains has only a small effect on *K^i^_f_* [19]. A larger uncertainty of our calculation was related to OGxRG sequences. Unlike other high and medium affinity sites, the corresponding dissociation constant *K_d_*(OGxRG) = 4.6 µM, was measured for a shortened peptide [40]. Based on the results of [19], one terminal triplet of this peptide may interact with HSP47, potentially affecting the value of the dissociation constant. We, therefore, calculated *δ*(Δ*G*) and *δT* also assuming *K_d_*(OGxRG) = 0.94 µM, which was reported for a longer peptide with the PGxRG recognition sequence [40].
